# Evaluation of Local Application of Glucosamine Sulfate and Chondroitin Sulfate on Temporomandibular Joint Response and Alleviation of Pain and Tension During the Functional Treatment of Skeletal Class II Patients: A Randomized Control Clinical Trial

**DOI:** 10.7759/cureus.34608

**Published:** 2023-02-03

**Authors:** Maria N. Alhayek, Ahmad S. Burhan, Mohammad Y. Hajeer, Fehmieh R. Nawaya, Ghaith F. Sahtout

**Affiliations:** 1 Department of Orthodontics, University of Damascus Faculty of Dentistry, Damascus, SYR; 2 Department of Pediatric Dentistry, Syrian Private University Faculty of Dentistry, Damascus, SYR

**Keywords:** cephalometric analysis, twin-block, soft-tissue tension, tmj distances, pain, chondroitin sulfate, glucosamine sulfate, functional treatment, skeletal class ii

## Abstract

Objective: This study was conducted to assess the effects of applying a gel of combined glucosamine sulfate and chondroitin sulfate on the temporomandibular joint (TMJ) area in patients with skeletal Class II malocclusion treated by removable functional appliances in terms of TMJ internal proportions, levels of pain, and tension.

Materials and Methods: The study included 36 patients aged 10-13 years with skeletal Class II malocclusion due to retrusion of the mandible characterized by: 4-8 degrees of the sagittal skeletal discrepancy (ANB) angle, 4-7 mm of overjet, 72-76 degrees of the sagittal mandibular positioning (SNB) angle, and a bone maturity stage located at pubertal growth spurt. Patients were distributed to the experimental group (Twin-Block appliance + Jointance® gel) or the control group (conventional treatment with the Twin-Block appliance). An allocation ratio of 1:1 was employed. Pre- and post-treatment digital lateral cephalometric radiograms were taken, and the TMJ joint spaces were measured using the Viewbox software (dHAL Software, Kifissia, Greece). The pain and discomfort levels were evaluated using a questionnaire with a four-point Likert scale at three assessment times.

Results: The anterior and posterior glenoid and anterior condylar distances to the pterygoid vertical (PTV) reference plane significantly decreased after treatment (p<.001), and the anterior joint space decreased significantly (p<.001). In contrast, the superior distance of the condyle to the Frankfort horizontal reference plane increased significantly after treatment, and the same results were found for the posterior and superior joint spaces (p<.05). There were no significant differences between the two groups in the evaluated linear variables. No significant differences were found when comparing pain and tension levels between the two groups at each assessment time. A gradual decrease in pain and tension levels was observed between the three evaluation times in both groups.

Conclusions: A combination of glucosamine sulfate and chondroitin sulfate did not affect the temporomandibular joint spaces, pain, and tension levels in patients with skeletal Class II malocclusions treated by removable functional appliances.

## Introduction

Class II malocclusion is one of the most common orthodontic problems, affecting approximately one-third of the population [1]. Mandible retrusion is the foremost important reason for its occurrence; thus, stimulating mandibular growth is the best treatment for these cases, and various functional appliances have been designed to correct this type of skeletal and occlusal asymmetry [2].

The forwarding position of the mandible stimulates cellular and molecular in the temporomandibular joint (TMJ), which leads to condylar and glenoid fossa growth [3]. The mechanical forces produced by forwarding the mandible stimulate the cellular and molecular responses in the condyles, which induce changes in chondrocytes and lead to osteogenesis [4].

Throughout the treatment with functional devices, the patient may feel some discomfort in the TMJ area, pressure around the teeth, tension in the oral muscles, and pain in the TMJ and, probably, the masticatory muscles [5]. Haynes considered pain as the main reason for patients to stop orthodontic treatment [6]. To decrease pain and discomfort and enhance patient compliance, many suggestions have been made [5]. El-huni et al. concluded that modifying the dimension, size, and color of the appliance can decrease the level of pain and discomfort and improve the cooperation of the patients [7]. However, pain and discomfort may be due to the temporomandibular joint response to the functional appliance, which increases the inflammatory factors [5]; therefore, pain will be the primary feeling [6]. Some recent studies have shown the ability to reduce pain and improve function in joint osteoarthritis when using the combination of glucosamine sulfate (GS) and chondroitin sulfate (CS) [8]. Other studies have shown its effectiveness in reducing temporomandibular disorders (TMD)-related pain and improving the maximum mouth opening [9,10]. 

In addition to their analgesic and anti-inflammatory effects, Barley et al. used these materials in an experimental study on animals undergoing functional appliances. These materials successfully modified condylar growth and facilitated mandibular forward positioning. They found a biochemical stimulation with excellent safety and minimal side effects [11]. The study gave promising results in enhancing the biological response to functional device therapy by using GS and CS, which was positively reflected in the growth of the mandible.

The stimulation of condylar growth during functional treatment of skeletal Class II growing patients has been under evaluation by several researchers in the last few years. Abdulhadi et al., in a randomized controlled trial (RCT), showed that laser irradiation on the TMJ area in patients undergoing functional appliance treatment shortened the treatment time and improved condylar bone growth [12]. Another study by Namera et al. found great effectiveness of low-intensity pulsed ultrasound in shortening the functional treatment period and enhancing mandibular growth [13]. On the contrary, Mohammad found no effect of laser application on skeletal changes after functional treatment [14].

Until now, no clinical study has been conducted to evaluate the effect of applying a combination of GS and CS on the TMJ area in relieving possible pain and discomfort during functional treatment of skeletal Class II problems and enhancing bone remodeling and condylar growth. Therefore, the current study aimed to assess the effect of treatment with the Twin-Block appliance in conjunction with a local application of GS and CS on the TMJ response and the levels of pain and tension during functional correction of skeletal Class II patients.

## Materials and methods

Study design and settings

This study was a two-arm parallel-group randomized controlled clinical trial with a 1:1 allocation ratio. The study was conducted at the Department of Orthodontics, Faculty of Dentistry, Damascus University, Damascus, Syria. Ethical approval was obtained from the Research Ethics Committee of the Faculty of Dentistry, University of Damascus (Approval number: UDDS-648-18062019/SRC-3034), and this RCT was registered in the German Clinical Trial database (DRKS00028975).

Sample size estimation

The sample size was estimated using G*Power 3.1.6 program (the Heinrich-Heine University, Dusseldorf, Germany), with the following assumptions: the 2-sample t-test was applied, the statistical power of 95% and a significance level of 0.05 was assumed, the changes in SNB angle was used to calculate the effect size depending on data from Giuntini et al.'s study [15], the effect size was 1.39, and the required sample size was 15 patients in each group; the number was increased to 18 patients in each group to compensate for withdrawal if it occurs.

Patients' recruitment and follow-up

The participants were selected from the patients registered in the pending records at the Department of Orthodontics, Damascus University. A total of 52 patients with a primary diagnosis of Class II division I malocclusion were recalled for further examination. Data were collected from November 2019 to October 2021. Inclusion criteria were: skeletal Class II malocclusion due to retrusion of the lower jaw (the sagittal mandibular positioning angle between 72º and 76º), sagittal skeletal discrepancy (ANB) angle between 4º and 8º, vertical growth pattern (Bjork sum: 396º ±6º), overjet between 4 and 7 mm, overbite between 1 mm and 4 mm, and bone maturity at pubertal growth spurt peak determined by hand-wrist radiograph according to the Grave and Brown method [16]. Exclusion criteria were: extreme vertical or horizontal growth patterns, transverse skeletal disorders such as patients with mandibular deviation more than 2 mm from the facial midline, previous orthodontic treatment, systemic diseases, congenital disability, trauma or previous operations, poor oral hygiene, and allergy to any component of the gel used. The pathological history was recorded, intra-oral and extra-oral clinical examination was conducted, and the diagnostic form for each patient was filled. A wrist-hand radiographic image was taken to determine the bone maturity, and signed consent of the patient's parents was taken to participate in the study after they were informed of the research steps and the nature of the treatment.

Randomization and allocation concealment

Microsoft Excel 2016 (Microsoft Corporation, Redmond, Washington, United States) was used to generate a random list to distribute the patients to the experimental group or the control group with an allocation ratio of 1:1. The allocation concealment was done using sequentially numbered, sealed envelopes that were opened at the start of the treatment. 

Intervention

The Experimental Group: Twin-Block Plus the Jointance® Gel (TB+J)

**Figure 1:**
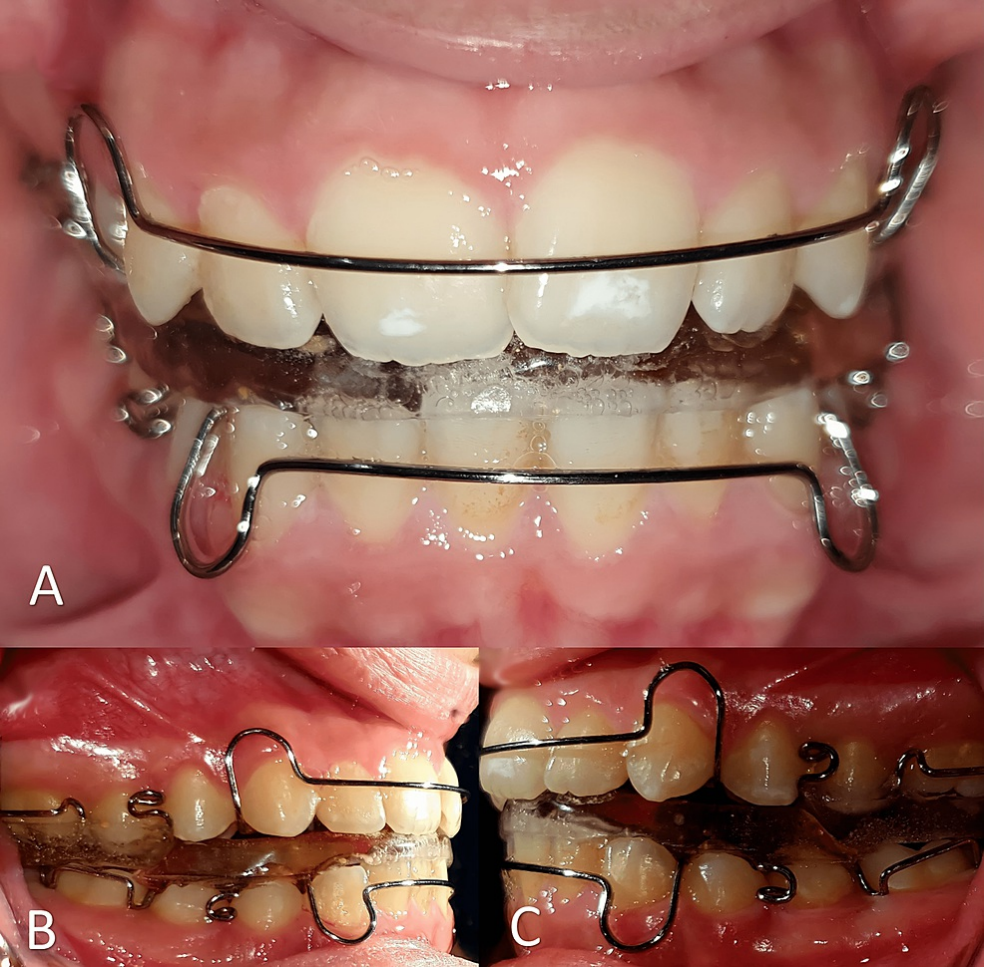
The Twin-Block appliance used in the current trial; (A) Frontal view, (B) Right side, (C) Left side.

Patients in this group were treated with a Twin-Block appliance with the application of the Jointace® gel. Alginate impressions of the jaws were taken in addition to the functional plate wax bite. The functional bite was taken by asking the patient to forward the mandibular to the edge-to-edge position, with a thickness of 2 mm between the incisors. The coincidence of the chin midline with the facial midline was considered when designing the Twin-Block device. The device consisted of an upper movable plate with a central expander and Adams clasps on the permanent first molars and acrylic blocks on the first premolars and first molars, and a lower movable plate with Adams clasps on the first molars and acrylic blocks on the premolars, in addition to an extension of acrylic to cover the anterior teeth (Figure 1). All appliances were constructed by the same orthodontic technician using self-curing acrylic (Orthocryl®; Dentaurum GmbH & Co., Ispringen, Germany).

The patients were instructed to wear the appliance at all-time except meal times, and their parents were asked to remind the patient about this. They were reviewed at intervals of three weeks, and the upper midline expansion screw was turned once a week (0.2 mm) until the necessary transverse expansion was achieved. The active phase of treatment continued until the overjet reached 0-1.5 mm in the centric occlusion (i.e., overcorrection). The active treatment was followed by a removable anterior inclined plane to maintain the correction in the incisor's relationship and support the buccal segments settling fully into occlusion. After the retention period, 25 patients needed fixed appliances, which were applied at the Department of Orthodontics, Damascus University; six patients did not need fixed appliances, and five dropped out. All patients were followed up until the end of active and retention treatment by the same orthodontist (MNA).

Gel application: A topical gel, Jointace® Gel (Vitabiotics Ltd., London, England), was used. This gel consisted of a dual blend of soluble GS and CS, essential oils extracts such as ginger, fennel, soothing menthol, and other inactive ingredients to help the gel absorb through the skin. All of these ingredients have been dermatologically tested, and it is applicable for adults and children over 10 years old, according to the manufacturing company. The patients in this group were instructed to apply the gel on the skin in the area of the TMJ, in front of the earlobe, bilaterally. They were asked to apply it twice a day according to the protocol recommended by the manufacturer. They were warned about not getting the gel in contact with injured or scratched skin or coming close to the eye. The gel was applied once in the morning and once in the evening.

The Control Group: The Traditional Treatment Plus the Placebo Gel (TB+P)

In this group, the patients were treated with a Twin-Block plus placebo. The placebo gel consisted of inactive ingredients such as ethanol, glycerin, carbomer, mineral oil, and menthol. This gel was manufactured by a pharmacologist in a private pharmaceutical laboratory, with the same characteristics as Jointace gel, such as color and smell. The two gels used had the same outer shell, covered with a white cap. The active and retention protocols were similar to those in the Experimental group.

Outcome measures

Cephalometric Measurements

Pre- and post-treatment digital lateral cephalometric radiograms were taken in central occlusion positions for every patient using the radiographic apparatus (Soredex Cranex D; Danaher Corporation, Washington, D.C., United States), with the same settings (energy of 70 kV, 10 mA and exposure time of 0.6 seconds). The distance of 130 cm from the x-ray source to the sagittal plane of the head and 15-20 cm from the sagittal plane of the head to the film. Cephalometric images were evaluated using Viewbox version 4.0.1.6 (Released 2012; dHAL Software, Kifissia, Greece). The cephalometric analysis consisted of seven linear variables that described the TMJ distances (Table 1). The definitions of the variables were taken from the study of Shotell [17] and Miao et al. [18]. The same radiographic apparatus took all cephalometric radiographs in the same settings. Therefore, the magnification factor was almost close in all patients and was found to be 14%.

**Table 1:** Definitions of the linear measurements used in the current work. * The definitions of variables were taken from Shotell, 2014 [17]; ^† ^The definitions of variables were taken from Miao et al., 2018 [18]. TMJ: temporomandibular joint; PTV: pterygoid vertical; FH: Frankfort horizontal

TMJ linear measurements	Definition
Anterior glenoid fossa horizontal position (AG)^*^	Distance from the most prominent point on the anterior wall of the glenoid cavity to the PTV
Posterior glenoid fossa horizontal position (PG)^*^	Distance from the most prominent point on the posterior wall of the glenoid cavity to the PTV.
Anterior condyle horizontal position (AC)^*^	Distance from the most prominent point on the anterior surface of the mandibular condyle to the PTV.
Superior condyle vertical position (SC)^*^	Distance from the highest point on the upper surface of the mandibular condyle to the FH line.
Anterior joint space (AJS) ^†^	Space between the anterior surface of the condyle and the anterior wall of the glenoid cavity.
Posterior joint space (PJS) ^†^	Space between the posterior surface of the condyle and the posterior wall of the glenoid cavity.
Superior joint space (SJS) ^†^	Space between the upper surface of the condyle and the upper wall of the glenoid cavity.

Pain and Feeling of Tension of The Oral Muscles Questionnaire

The pain and discomfort levels were evaluated using a questionnaire consisting of two questions; the first was about the sensation of pain, and the second was about the feeling of tension in the peri-oral muscles. The questionnaire was accompanied by a four-point Likert scale as follows: 0 indicated that there was no feeling of pain or tension at all, 1 indicated that there was a little feeling of pain or tension, 2 indicated that there was a moderate feeling of pain or tension, and 3 indicated that there was a very much feeling of pain or tension. The questionnaire points were explained to each patient and his/her parents. They were asked to complete the questionnaire at the following assessment times: one week (T1), two weeks (T2), and three months (T3) following appliance insertion. 

Error of method

Thirty cephalometric radiograms were randomly selected and re-evaluated by the principal researcher (MA) one month later. The differences between the two readings were compared using paired t-tests to detect any systematic error. The random errors were evaluated using the intraclass correlation coefficients (ICCs).

Statistical analysis

The statistical study was carried out using IBM SPSS Statistics for Windows, Version 23.0 (Released 2015; IBM Corp., Armonk, New York, United States). The normality of data was distributed according to the Shapiro-Wilk test. Gender distribution differences between the two groups were evaluated using the Chi-square test. The differences in age between the two groups were evaluated using an independent sample t-test. The differences in the seven linear variables were compared between the two groups using an independent sample t-test. The TMJ dimension changes before and after treatment among the two groups were evaluated using paired sample t-test. Non-parametric tests were used to evaluate the differences in pain and tension levels within each group and between the two groups because the patient’s answers to the questionnaire were considered ordinal parameters (0,1,2,3). Therefore, the differences in pain and tension levels between the two groups at each assessment time were evaluated using the Mann-Whitney test. The changes in pain and tension levels over the three assessment times within each group were evaluated using the Friedman test and the Wilcoxon signed-rank tests as post-hoc tests for pairwise comparisons.

## Results

Patients' follow-up and baseline sample characteristics

**Figure 2:**
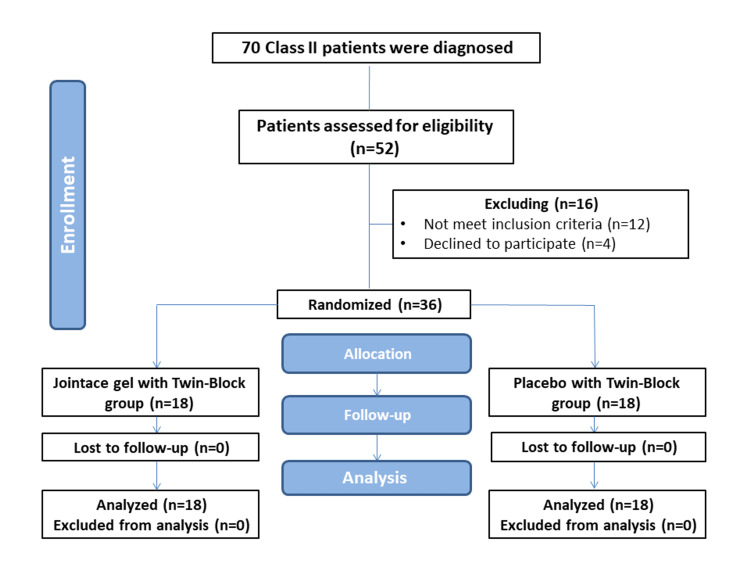
The Consolidated Standards of Reporting Trials (CONSORT) flow diagram of patients' recruitment, follow-up, and entry into data analysis

The Consolidated Standards of Reporting Trials (CONSORT) flow diagram illustrates the patient's flow through the study (Figure 2). Thirty-six patients were randomized into either the Experimental group or the Control group. No dropouts occurred. Complete follow-up was done for all patients. Therefore, an intention-to-treat analysis was performed. The descriptive statistics of the sample regarding gender and age are given in Table 2. No significant differences were observed in gender distribution (p=0.095) and ages (p=0.125) between the two groups.

**Table 2:** Baseline sample characteristics * Statistical significance in gender distribution; † Statistical significance for mean age between the two groups. SD: standard deviation; Max: maximum; Min: minimum

Study group	Patient’s gender	Number of patients	Age (years)				P-value^*^	P-value ^†^
			Mean	SD	Max	Min		
Experimental (Jointace^®^) group	Male	7	12.42	0.78	14	12	0.095	0.125
	Female	11	12.18	0.56	13	11.50		
	Total	18	12.24	0.64	14	11.50		
Control (Placebo) group	Male	12	11.95	0.58	12.90	11		
	Female	6	11.75	0.27	12	11.50		
	Total	18	11.88	0.50	12.90	11		
Total	Male	19	12.13	0.68	14	11		
	Female	17	12.02	0.51	13	11.50		
	Total	36	12.08	0.60	14	11		

Error of method

No systematic errors were found for any variables, and the ICC values were close to one (Table 3), which meant the calculated measurements were consistent between the first and second readings. Therefore, one reading was used in the data analysis.

**Table 3:** The differences between the first and the second readings and the ICC values for each assessed variable. *Paired sample t-test; †P-value of the ICCs AG: anterior glenoid fossa horizontal position; PG: posterior glenoid fossa horizontal position;  AC: anterior condyle horizontal position; SC: superior condyle vertical position; AJS: anterior joint space; PJS: posterior joint space; SJS: superior joint space; ICC: intraclass correlation coefficients

variable	Cephalometric count	First reading		Second reading		p-value^*^	ICC value	p-value^†^
		Mean	SD	Mean	SD			
AG	30	23.22	1.63	23.1	1.18	0.62	0.924	<0.001
PG	30	35.62	2.07	35.58	1.79	0.86	0.944	<0.001
AC	30	25.16	1.77	25.25	1.79	0.67	0.933	<0.001
SC	30	0.61	1.17	0.79	0.91	0.4	0.880	0.002
AJS	30	1.81	0.42	1.79	0.36	0.88	0.925	<0.001
PJS	30	2.17	0.48	2.23	0.44	0.45	0.927	<0.001
SJS	30	2.32	0.24	2.36	0.23	0.34	0.926	<0.001

TMJ changes

There were similar changes among the two groups before and after treatment (Table 4). The anterior and posterior glenoid distances to the pterygoid vertical (PTV) were significantly decreased by a mean of 0.32 mm and 0.38 mm in the Experimental group and 0.19 mm and 0.49 mm in the Control group, respectively. The anterior condylar distance to the PTV plane significantly decreased after treatment by a mean of 0.68 mm in the Experimental group and 0.47 mm in the Control group (p<0.001). The superior condylar distance to Frankfort horizontal (FH) plane increased significantly in the two groups. There was a mean increase of 0.2 mm in the Experimental group (p=.015) and 0.17 mm in the Control group (p=.022). There was a significant decrease in the anterior joint space after treatment in the Experimental and the Control groups by a mean of 0.36 mm and 0.31 mm, respectively (p<0.001). In the Experimental group, there was a significant increase by a mean of 0.58 mm and 0.3 mm in the posterior and superior joint spaces, respectively (p<0.001), while in the Control group, there was a significant increase by a mean of 0.49 mm and 0.34 mm in the posterior and superior joint spaces, respectively (p<0.001). There were no significant differences when comparing the changes in the TMJ variables between the two groups.

**Table 4:** Descriptive statistics of the measured variables at the two assessment times in both groups and the P-values of intra-group significance testing *Paired sample t-test; ^†^Independent sample t-test AG: anterior glenoid fossa horizontal position; PG: posterior glenoid fossa horizontal position;  AC: anterior condyle horizontal position; SC: superior condyle vertical position; AJS: anterior joint space; PJS: posterior joint space; SJS: superior joint space

Studied variable	Experimental (Jointace^®^) group					Control (Placebo) group					Diff between groups
	Before		After		P-value^*^	Before		After		P-value^*^	P-value ^†^
	Mean	SD	Mean	SD		Mean	SD	Mean	SD		
AG	23.33	1.98	23.01	2.03	<0.001	23.46	1.85	23.27	1.84	<0.001	0.72
PG	36.21	1.86	35.83	1.89	<0.001	36.22	1.62	35.73	1.64	<0.001	0.26
AC	25.04	1.78	24.36	1.9	<0.001	25.20	1.71	24.73	1.64	<0.001	0.39
SC	0.70	0.95	0.9	1.15	0.015	0.77	0.90	0.94	1.15	0.022	0.75
AJS	1.70	0.48	1.34	0.45	<0.001	1.76	0.51	1.45	0.45	<0.001	0.38
PJS	2.27	0.50	2.85	0.5	<0.001	2.23	0.43	2.72	0.48	<0.001	0.39
SJS	2.62	0.57	2.92	0.56	<0.001	2.48	0.47	2.81	0.63	<0.001	0.690

Pain and tension

No significant differences in pain and tension levels were found between the two groups at each assessment time (p>0.05; Table 5). The pain and tension levels gradually decreased from the first assessment time to the last among the assessment times in the two groups. In the Experimental group, the mean pain levels were 1.5, 0.33, and 0.06 points at T1, T2, and T3, respectively. At the same time, the mean tension levels were 1.94, 0.94, and 0.33 points at T1, T2, and T3, respectively. In the Control group, the mean pain levels were 1.67, 0.17, and 0.11 points at T1, T2, and T3, respectively, while the mean levels of tension were 2.22, 1.06, and 0.28 points at T1, T2, and T3, respectively. There were significant differences between all assessments time in each group (p<0.05; Table 6)

**Table 5:** Descriptive statistics of the levels of pain and tension at three assessment times and the p-values of significance testing *Mann-Whitney test

Question	Period	Experimental (Jointace^®^) group					Control (Placebo) group					Diff between groups
		Mean	SD	Median	Q1	Q3	Mean	SD	Median	Q1	Q3	P-value^*^
Pain	T1	1.5	0.61	1	1	2	1.67	0.76	1.5	1	2	0.093
	T2	0.33	0.48	0	0	1	0.17	0.38	0	0	0	0.274
	T3	0.06	0.23	0	0	0	0.11	0.32	0	0	0	0.058
Tension	T1	1.94	0.8	2	1	3	2.22	0.8	2	1.75	3	0.297
	T2	0.94	0.63	1	0.75	1	1.06	0.63	1	1	1.25	0.598
	T3	0.33	0.48	0	0	1	0.28	0.46	0	0	1	0.721

**Table 6:** Descriptive statistics of the changes in the levels of tension and pain in the two groups, along with the P-values of intragroup and intergroup significance testing * Friedman test; ^†^Pairwise comparisons using Wilcoxon test

Question	Experimental (Jointace^®^) group				Control (Placebo) group			
	Period	P-value^†^	chi value	P Diff ^*^	Period	P-value^†^	Chi value	P Diff ^*^
Pain	T1-T2	0.002	26.85	<0.001	T1-T2	0.001	25.200	<0.001
	T1-T3	<0.001			T1-T3	<0.001		
	T2-T3	0.013			T2-T3	0.029		
Tension	T1-T2	0.001	27.74	<0.001	T1-T2	0.001	32.375	<0.001
	T1-T3	<0.001			T1-T3	<0.001		
	T2-T3	0.002			T2-T3	<0.001		

## Discussion

This is the first clinical trial to evaluate the effects of a topical bio-absorbable material applied in the TMJ area on condylar growth and the associated perceptions of pain and tension of the perioral muscles when treating skeletal Class II patients with removable functional appliances.

TMJ changes

The anterior joint space was significantly decreased after treatment in the Experimental and the Control groups by a mean of 0.36 mm and 0.31 mm, respectively (p<0.001). In the Experimental group, there was a significant increase by a mean of 0.58 mm and 0.3 mm in the posterior and superior joint spaces, respectively (p<0.001). While in the Control group, there was a significant increase by a mean of 0.49 mm and 0.34 mm in the posterior and superior joint spaces, respectively (p<0.001). The changes in the joint spaces could have been due to the anterior and inferior movement of the condyles after forwarding the mandible by the Twin-Block appliance [19,20]. These results agreed with those of Elfeky et al.'s study, which indicated that the superior and posterior joint spaces were increased when using the Twin-Block appliance with a decrease in the anterior joint space [20]. They also agreed with the findings of Bowen et al.'s study, which evaluated the effect of Twin-block on TMJ by using cone-beam computed tomographic (CBCT) images [21]. in contrast, these results disagreed with the results of Kinzinger et al.'s study, which indicated no changes in the position of the condyles in the glenoid fossa after treatment by fixed functional treatment [22]. The disagreement may be due to the use of magnetic resonance imaging (MRI) in their study and the used of fixed appliances.

The anterior and posterior glenoid distances to PTV were significantly decreased by a mean of 0.32 mm, and 0.38 mm in the Experimental group and 0.19 mm, and 0.49 mm in the Control group, respectively. The anterior condylar distance to PTV significantly decreased after treatment by a mean of 0.68 mm in the Experimental group and 0.47 mm in the Control group (p<0.001). These findings may be explained by the anterior movement of the condyles, which caused resorption in the anterior wall of the glenoid fossa and apposition in the posterior wall [23]. These findings agreed with those of LeCornu et al., which showed that the Herbst appliance might cause resorption in the anterior wall of the glenoid fossa and apposition in the posterior wall [23].

The superior condylar distance to FH increased significantly in the two groups. There was a mean increase of 0.2 mm in the Experimental group (p=0.015) and 0.17 mm in the Control group (p=0.022), in agreement with Bowen et al.'s study, which noted that the height of the condyles increased after functional treatment by Twin-Block due to the bone apposition on the superior surface of the condyles [21]. In contrast, these results disagreed with the study of Parvathy et al., which found a decrease in the condyles' height after treatment with a Twin-Block appliance [24]. The disagreement may be due to the use of CBCT in their study, which is considered more accurate than a cephalometric radiogram in evaluating TMJ.

No statistically significant differences were found when comparing all previous variables between the two groups (p>.05). Thus, the local application of Jointace gel did not affect the changes of TMJ spaces during skeletal Class II functional treatment [19,20]. No studies were conducted on humans to evaluate the effect of GS and CS in combination with functional appliances. Only one experimental study was found in the literature, showing an effect of the previous materials on cartilage volume in rats [11], but its results were inconsistent with this study's results. The difference may be attributed to the nature of the studied sample and the GS and CS delivery method.

Levels of pain and tension

The pain and tension levels gradually decreased from the first assessment time to the last one among the assessment times in the two groups. In the Experimental group, the mean pain levels were 1.5, 0.33, and 0.06 points at T1, T2, and T3, respectively. On the other hand, the mean tension levels were 1.94, 0.94, and 0.33 points at T1, T2, and T3, respectively. In the Control group, the mean pain levels were 1.67, 0.17, and 0.11 points at T1, T2, and T3, respectively. On the other hand, the mean tension levels were 2.22, 1.06, and 0.28 points at T1, T2, and T3, respectively. There were significant differences between all assessments time in each group (p<0.05). The pain and tension may gradually decrease because of the masticatory muscle's reaction to massage in the application area [25], in addition to the patient’s adaption to pain and discomfort when the treatment progress [26,27]. These results agreed with those of Al-Ahmed et al.'s study, which noted that the pain and discomfort occurred in the first two weeks after the application of the Herbst appliance and then decreased within three months [26]. They also agreed with Idris et al.'s study, which concluded that pain and tension started in the first two weeks and then gradually decreased due to the adaption of the patients [5].

No statistically significant differences in pain and tension levels were found between the two groups at each assessment time (p>0.05). Therefore, applying the gel did not affect the pain and tension sensations resulting from the functional appliance. According to Yang et al., the combination of GS and CS needs a period of 6-12 months to affect the nature of the joint and relieve pain in TMJ disorders [28]. Therefore, this result may be explained by the short period of pain assessment and the patients' adaption to all previous studies that evaluated the effect of GS and CS on pain in the cases of TMJ disorders. However, none of these studies evaluate the effect of these bio-materials on pain and tension induced by functional treatment. The systematic review conducted by Ding et al. showed that the Class II functional treatment might cause TMJ disorders symptoms at the active treatment phase [29]. Therefore, comparing this study's results and the results of TMDs studies may be acceptable. This study agreed with Damlar et al.'s study, which showed a similar reduction in pain when comparing the use of GS and CS with tramadol in patients with TMJ disorders and pain [9]. This also agreed with the results of the study of Cahlin and Dahlström conducted on patients with disorders and pain in the TMJ and showed no difference when comparing a group given oral glucosamine and a group given a placebo drug [30]. In contrast, the current results disagreed with those of Thie et al.'s study, which showed pain reduction in TMJ disorders in patients treated with GS compared to patients treated with ibuprofen [10]. The disagreement might be due to the difference in the evaluation method of the effect of GS and CS on pain.

Limitations

There are a few limitations of this study. First, there was no group of untreated patients in the current trial to evaluate the normal growth of the condyle. Second, the TMJ internal distances were evaluated using two-dimensional cephalometric radiograms only; i.e., no CBCT or MRI images were used. Further studies using three-dimensional imaging modalities may be needed to confirm the results of this study. Third, patient commitment to using the gel and applying it to the required region was not assessed, and we assumed their use was regular and according to the given plan. Finally, the sample size may be small, and future studies with large sample sizes may be needed to generalize the results of this study.

## Conclusions

The anterior joint space was decreased, and the posterior and superior joint spaces were increased after treatment in both groups. The differences between the two groups were insignificant. This indicates that the local application of GS and GS did not affect the TMJ space changes after functional treatment. No differences were found in the pain and tension levels between the two groups, indicating no effect of the used gel on the perceptions of pain and tension in patients with skeletal Class II malocclusions treated by removable functional appliances.
